# Optical Automatic Contour Tracing (O‐ACT) – A novel optical image‐guided contour tracing method for electron beam shaping

**DOI:** 10.1002/mp.17555

**Published:** 2024-11-29

**Authors:** James J. Sohn, Jeonghoon Park, Siddhant Sen, Tianming Wu, Matt Mielke, Siyong Kim

**Affiliations:** ^1^ Department of Radiation and Cellular Oncology University of Chicago Chicago Illinois USA; ^2^ Department of Medical Physics Memorial Sloan Kettering Cancer Center Basking Ridge New Jersey USA; ^3^ Department of Psychology University of Illinois Urbana‐Champaign Champaign Illinois USA; ^4^ Department of Radiation Oncology Northwestern Memorial Hospital Northwestern University Feinberg School of Medicine Chicago Illinois USA; ^5^ Department of Radiation Oncology Virginia Commonwealth University Richmond Virginia USA

**Keywords:** electron applicator, electron cutout, electron therapy, magnification factor, skin cancer

## Abstract

**Background:**

Electron therapy, vital for treating skin lesions and superficial tumors, demands precise electron cutouts to ensure accurate dose delivery. Traditional manual tracing methods introduce uncertainties and inefficiencies, necessitating innovative solutions for custom block creation.

**Purpose:**

To introduce and validate the Optical Automatic Contour Tracing (O‐ACT) method, enhancing electron cutout generation's accuracy and efficiency through optical imaging and software automation.

**Methods:**

Utilizing a 3D‐printed holder, a centrally‐mounted charge‐coupled device (CCD) camera on the electron applicator secured two distinct perspective images of the skin's contour at varying heights. Through image binarization and skeletonization, we identified the clinical target contour's region‐of‐interests (ROIs) in each setup image. Employing distances from each ROI's center, assessed at 5‐degree intervals from both images, we reconstructed the target contour on the skin. The magnification factor, set at a 95 cm source‐to‐point distance, determined the final cutout shape. We crafted an in‐house software in MATLAB for camera calibration and image processing and juxtaposed our results against the standard clinical cutout from the treatment planning system (TPS) using a correlation coefficient based on masked binary images' mutual information. Additionally, we performed dosimetric evaluations using abstract shapes to compare O‐ACT with other methods.

**Results:**

Our methodology yielded cutout shapes exhibiting remarkable alignment with the TPS clinical cutout. O‐ACT demonstrated superior precision in generating cutout shapes that closely align with the contours in TPS, improving upon other methods in terms of adaptability to patient body shapes and contour accuracy. Dosimetric evaluations showed minimal differences between methods, with O‐ACT providing slightly more consistent results. Dose profile analyses in penumbra regions indicated O‐ACT's improved accuracy compared to conventional methods.

**Conclusions:**

Pushing the boundaries of traditional practices, our O‐ACT offers a more accurate, efficient, and reproducible method for custom electron cutout creation from clinical setup images. This innovation promises not only to streamline clinical workflow but also to potentially uplift clinical outcomes in radiation oncology by providing more accurate patient‐specific treatment accessary.

## 1 INTRODUCTION

Electron therapy is uniquely suited to treat skin lesions and superficially located tumors, while reducing potential damage to underlying tissues.^1^, ^2^, ^3^ This modality employs an electron beam, that is directed onto the target through an electron applicator. Mounted onto the outside of the gantry head, this electron applicator plays a critical role in shaping and steering the beam accurately toward the tumor.^4^ The use of electron therapy necessitates the use of beam shaping aperture, known as electron blocks (often called “cutout”), to confine the radiation dose to the treatment area.^5^ These blocks come in two primary forms: standard and custom. Standard electron blocks which are shaped as square, rectangular, or circular, are designed for regular and/or geometrically simple treatment areas. However, cancerous lesions often present irregular and complex shapes that necessitate the use of custom electron blocks typically made of Cerrobend.^6^ These blocks are specifically designed to match the physician's contour of the treatment area, offering a more personalized and targeted approach to treatment. This custom approach, while therapeutically beneficial, presents its own unique set of challenges.^4^, ^6^

Despite the benefits, the successful delivery of electron therapy is contingent on the accurate delineation of the treatment target, typically defined directly on the patient's skin during a clinical simulation. This conventional manual tracing method, while well established, is subject to significant uncertainties.^7^ The process of creating custom blocks often involves the physician drawing the contour directly on the patient's skin, after which a physicist or dosimetrist traces this contour back onto an empty transparent plate attached to the electron applicator. Therefore, this process is labor‐intensive and time‐consuming, two attributes that are suboptimal in the time‐sensitive clinical environment. Furthermore, the manual tracing process introduces an inherent level of uncertainty, which can impact the accuracy of dose delivery to the target and potentially affect treatment outcomes.

There were a couple of similar studies to address this cumbersomeness. Sidhu et al. developed a digital camera tray, that is installed in the upper wedge mount of Linac and made an irregularly shaped electron cutout from the acquired image.^8^ It had limited practicality only if the treatment field was planar and was not on highly sloping surfaces because of the single image acquisition at the fixed location. Bassalow and Sidhu extended the prior study by developing a digital camera mounting device to bring the camera focal spot to the Linac source position.^9^ It simplified the calculation of the magnification factor and could determine a more accurate cutout shape regardless of the slope of the patient's surface, even if the gantry cannot be at a clinical angle to bring the device to the source position. In this study, we developed an automatic contour tracing device, the Optical Automatic Contour Tracer (O‐ACT), using the stereotactic technique at two different locations, and introduced a fast and automatic contour digitization methodology using the O‐ACT. This approach utilizes a charge‐coupled device (CCD) camera mounted on the electron applicator, allowing for the optical capture of the projected contour. Complemented by in‐house developed software, this approach facilitates the automation of cutout preparation. Here we provide proof‐of‐principle for this framework and compare it with the conventional methods. Our goal is to transition from labor‐intensive, time‐consuming, and potentially error‐prone manual processes to more consistent, automated, and accurate ones using O‐ACT.

## 2 METHODS AND MATERIALS

### 2.1 Optical Automatic Contour Tracer (O‐ACT) and acquisition of contour images

**FIGURE 1:**
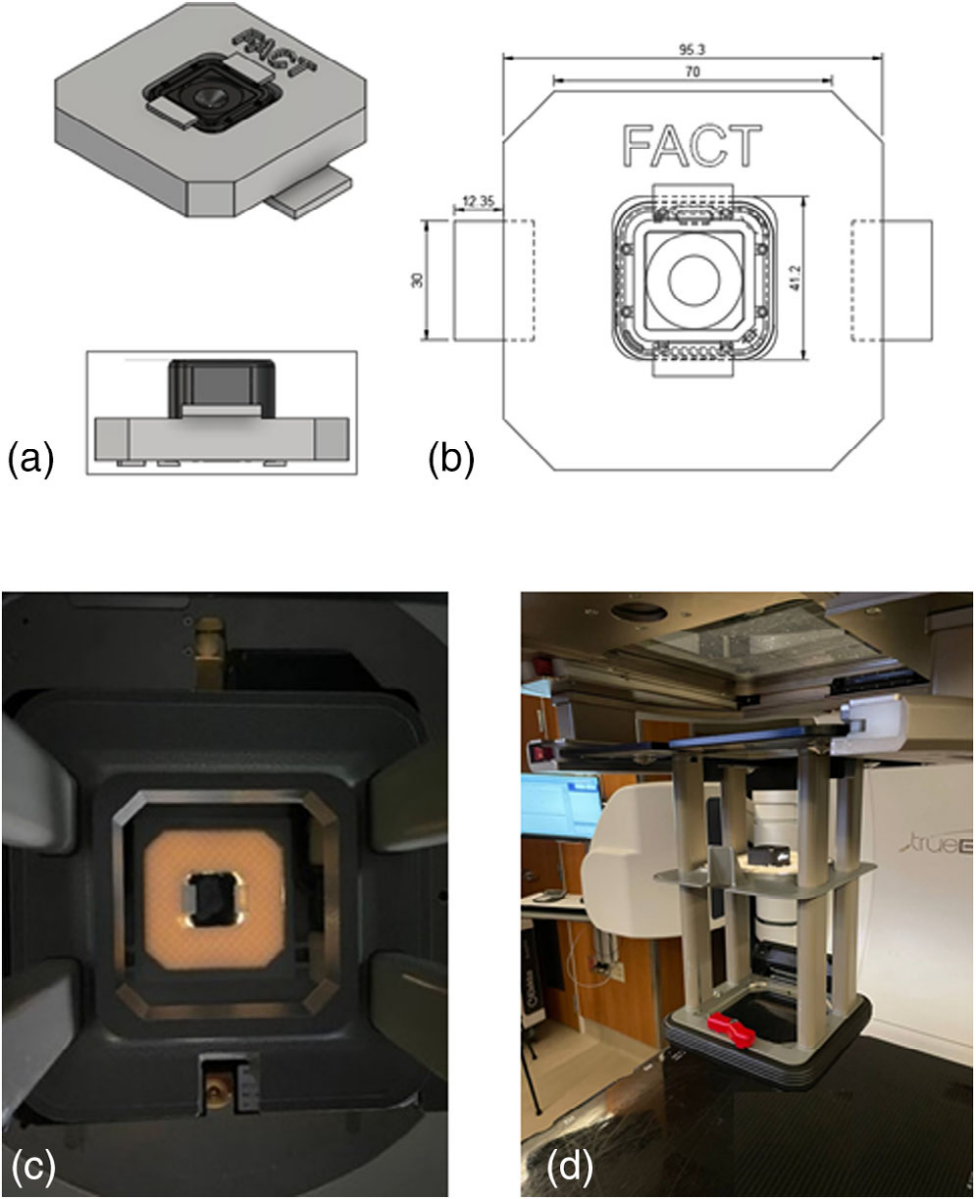
O‐ACT device design and implementation. (a) 3D design views of the camera holder, including dimensions and the screw for enhanced stability, and 3D renderings showing the camera placement and screw integration. (b) Actual implementation of the O‐ACT device, showcasing its mounting on the electron applicator. (c and d) Close‐up views of the device mounting at first and second scraper in the electron applicator. O‐ACT, Optical Automatic Contour Tracing.

The O‐ACT consisted of an optic camera (a GoPro HERO4 Session camera in this study—GoPro Inc., San Mateo, CA, USA) and a 3D‐printed camera holder.^10^ The camera holder was designed to be mounted onto an electron applicator. Specifically, it could be fixed to any of two scrapers of the applicator, as shown in Figure 1. Once connected to the applicator, it could provide still photos of the original contour. Equipped with a focal length of 26 mm, the camera provided an approximately 80‐degree field of view (FOV), enabling comprehensive coverage of every contour within the largest electron applicator dimension in our clinic (25 × 25 cm^2^). The camera was in a cube shape measuring 3.8 × 3.8 × 3.6 cm^3^ and weighing 74 g only. With a high resolution of 5 megapixels (2,720 × 2,040) in medium FOV mode, the camera provided detailed images of the contour.

### 2.2 Magnification calculation

**FIGURE 2:**
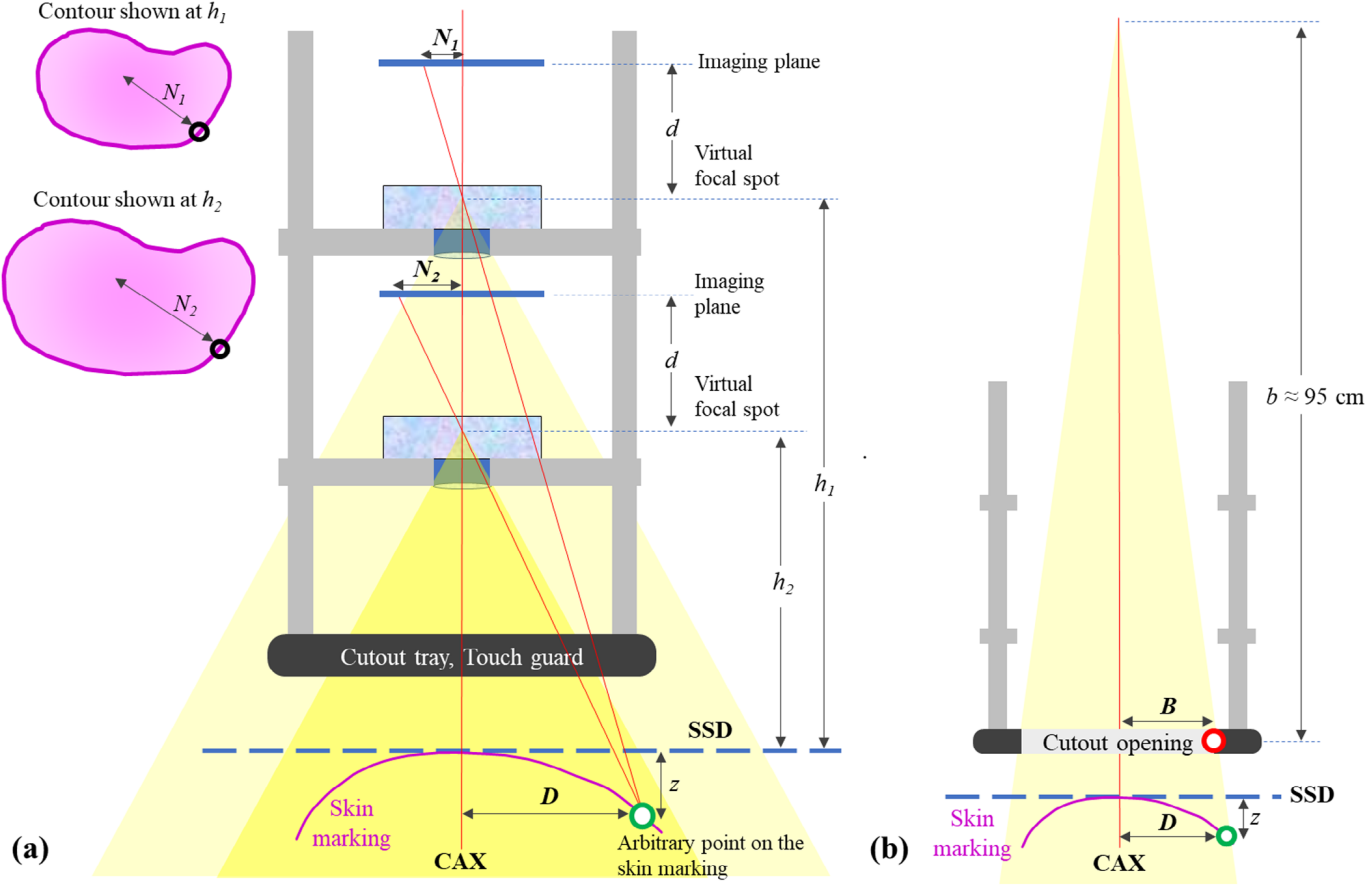
Methodology of O‐ACT for determining the magnification factor and the cutout coordinate using two photos taken at pre‐established heights within the electron applicator: Schematics for (a) Calculation of the magnification factor (b) Calculation of the cutout coordinate. O‐ACT, Optical Automatic Contour Tracing.

In our robust imaging configuration, the magnification factor plays a pivotal role. Figure 2 represents our schematics for determining the magnification factor using two photos. These photos are captured at different, but pre‐established, heights in the electron applicator. The predetermined nature of these heights imbues our method with a sense of consistency and repeatability, alleviating concerns associated with variable camera positioning. The distance measurement algorithm by Hsu et al. was used to calculate the 3D coordinate of the target contours projected to the CCD camera.^11^

Given these fixed heights, denoted as *h*_1_ and *h*_2_, two images are invariably captured, producing contours *N*_1_ and *N*_2_, respectively. To elucidate the magnification, we derive it from the spatial relationships of these contours relative to their camera position.

Considering the principles of projection geometry, the following relationships can be established:

$$\frac{D}{h_{1}+z}=\frac{N_{1}}{d}\;\mathrm{and}\;\frac{D}{h_{2}+z}=\frac{N_{2}}{d}$$

where:

- *D* represents an arbitrary point on the skin marking.
- *z* as the known distance between the isocenter plane (i.e., 100 cm SSD plane in general) and the point *D*.
- *d* signifies the vertical distance between the camera's optic origin and the imaging plane.

Isolating *D* from each equation and setting them equal provides a means to express *N*_1_ in terms of *N*_2_ (or vice‐versa) as given in the Appendix, thereby elucidating the magnification relationship between them. This proportional relationship not only offers a methodical understanding of how the contour's dimensions transform with respect to the camera's position but also underscores the accuracy and reliability of our approach.

Furthermore, the geometric framework combined with the aforementioned relationships permits a rigorous extraction of the 3D coordinates (*x*, *y*, *z*) of the skin marking as below from the bi‐planar image projections of (*x*_1_, *y*_1_) and (*x*_2_, *y*_2_). Through this geometric framework and relationships, it becomes feasible to determine the precise coordinates of the contour within the treatment room.

$$x=\frac{x_{1}x_{2}\left(h_{1}-h_{2}\right)}{d(x_{2}-x_{1})}\;,y=\frac{y_{1}y_{2}\left(h_{1}-h_{2}\right)}{d(y_{2}-y_{1})},\;z=\frac{x_{1}h_{1}-x_{2}h_{2}}{x_{2}-x_{1}}$$

Then, a cutout coordinate (*x_B_*, *y_B_*) could be calculated finally from a 3D coordinate (*x*, *y*, *z*) as below when the *b* denotes the distance from the source to the cutout, typically 95 cm. The equation shows that the contour magnification factor is different at a different distance if the contour lies at the concave or convex surface. Note that the cutout coordinates could be calculated using only two projection images without the necessity to have the depth of the skin marking contour.

$$x_{B}=\frac{x\cdot b}{SSD+z}\;\mathrm{and}\;y_{B}=\frac{y\cdot b}{SSD+z}\;$$

### 2.3 Image processing

The images captured were subsequently downloaded onto a local computer for processing. In this step, MATLAB was used to crop the images, isolating the square region and encapsulating the contour. Considering that the camera captures color images, isolating the contour proved to be a challenge. To address this, the cropped images were subject to MATLAB's image processing functions to convert the image to grayscale, apply a Gaussian filter, binarize, and then skeletonize the image.^12^ The output was a binary image showcasing the contour in black against a white background, along with the image statistics. These images and their corresponding statistics objects were then processed further to obtain the individual coordinates of the contour and the geometric center of the contour. The contour coordinates were calculated by dividing each contour into 72 equal intervals of 5 degrees within a loop, saving each intersectional pixel coordinate from the center point. This action yielded the coordinates (*x*_1_, *x*_2_) and (*x*_2_, *y*_2_) of the contours at imaging planes 1 and 2, respectively. Following this, both coordinates were incorporated into the following formula to calculate the final contour coordinate (*x*_new_, *y*_new_):

$$x_{\mathrm{new}}=\left(\left(h_{1}+h_{2}\right)/\left(d_{\mathrm{sc}}\right)\right)\times \left(\left(x_{1}+x_{2}\right)/2\right)$$

$$y_{\mathrm{new}}=\left(\left(h_{1}+h_{2}\right)/\left(d_{\mathrm{sc}}\right)\right)\times \left(\left(y_{1}+y_{2}\right)/2\right)$$

**FIGURE 3:**
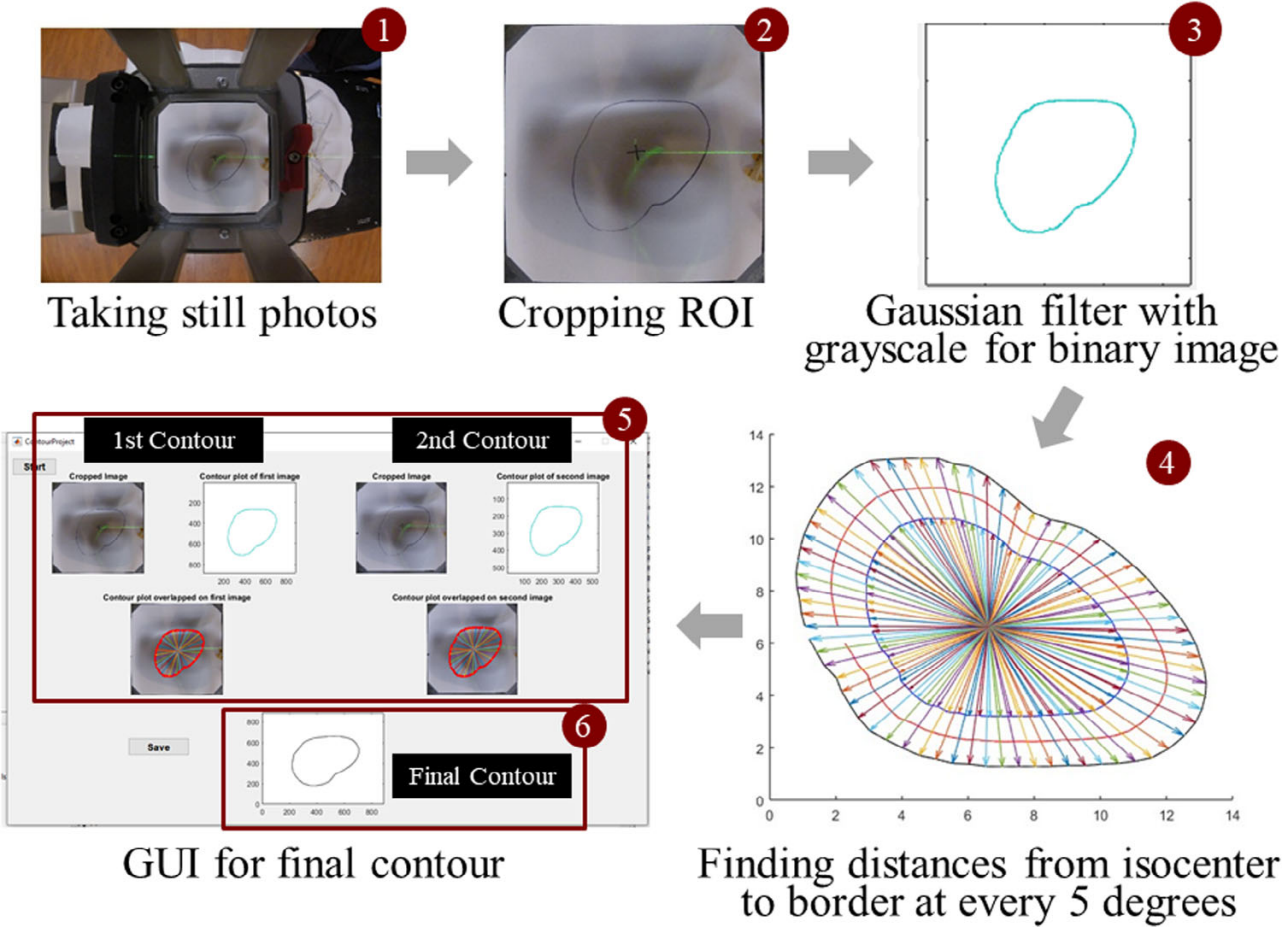
Workflow of the O‐ACT method. Starting with two photos from the electron applicator, images undergo processes including (1) ROI cropping, (2) grayscale conversion and Gaussian filtering, (3) center identification, (4) distance measurement from center to contour at 5‐degree intervals (adjustable), (5) comparison of two images to determine magnification factors, and (6) final shape output for electron cutout fabrication. O‐ACT, Optical Automatic Contour Tracing; ROI, region‐of‐interest.

The points obtained from this formula were stored in arrays for the final contour. This process ensured the accurate scaling of the new coordinates, resulting in an ideal contour that could be utilized as a template for creating a radiation therapy cutout. The final contour was plotted in MATLAB and made available for export in various file formats, including but not limited to PDF, JPG, and PNG. All procedures are illustrated in Figure 3.

### 2.4 Geometric validation

**FIGURE 4:**
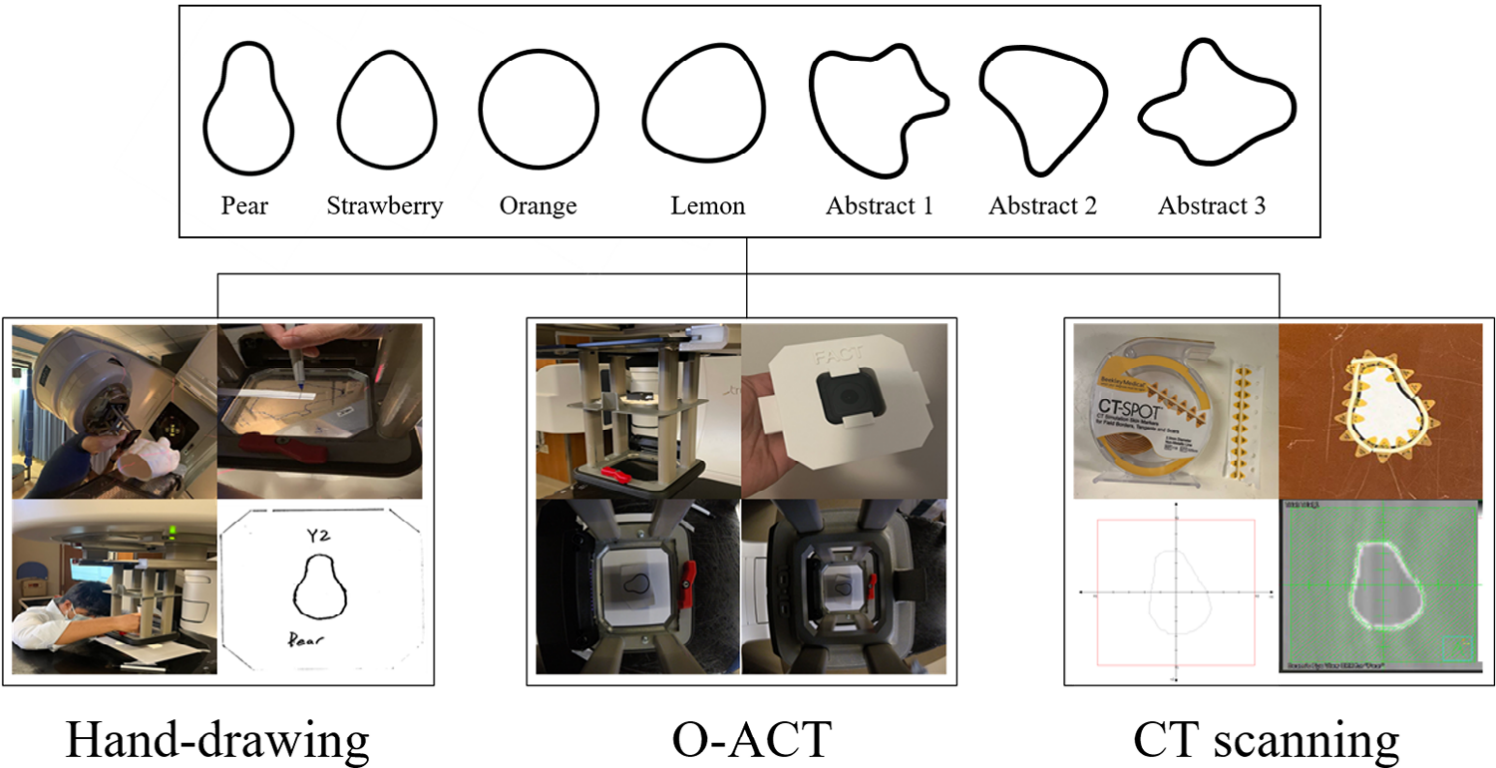
Comparative validation process showcasing three distinct methods: hand‐drawing illustrations, the O‐ACT approach, and CT scanning. The top panel displays various shapes used for validation. The subsequent images underline the processes and results associated with each method, underscoring the accuracy and versatility of the O‐ACT technique. CT, computed tomography; O‐ACT, Optical Automatic Contour Tracing.

The accuracy of our O‐ACT method was evaluated against two conventional methods commonly used in clinics, that is, (1) hand‐drawing manual technique and (2) computed tomography (CT) scanning‐based approach. Both of these traditional methods are regarded as standard practices in radiation therapy planning. As mentioned in the introduction section, in the hand‐drawing manual method, the contour is manually traced on a transparent plate placed on the electron applicator. In the CT scanning‐based approach, a string bearing radiation opaque wire is placed following the contour. The contour is determined using the wire shown in a beam's eye view digitally reconstructed radiography (DRR) image. To comprehensively assess the precision of each method, tests were conducted using four shapes in known geometry. Figure 4 illustrates the validation process using various shapes.

**FIGURE 5:**
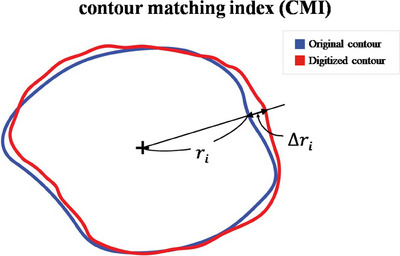
Visualization of the contour matching index (CMI) measuring the conformity of O‐ACT‐based method contours to original shapes. The blue line represents the original contour, and the red line indicates the digitized version. Discrepancies are quantified by the distance Δ𝑟_𝑖_ at regular 5‐degree intervals. O‐ACT, Optical Automatic Contour Tracing

Area ratio index (ARI) was introduced for quantitative analysis, which was the area ratio between the digitized contour for cutout generation and the original contour. Area calculation for ARI was based on the polygon method, also known as the Shoelace formula that calculated areas using the length of perimeter sides. The ARI provides a general assessment, allowing us to gauge how well the radiation dose distribution conforms to the target area. ARI values closer to one indicate better conformity. ARI is a similar concept to the conformity index (CI), which is commonly used in plan evaluation.^13^ Thus, it is simple and straightforward but can mislead the users when the excessive area is canceled out by shortage. To overcome this limitation, we defined a quantity, contour matching index (CMI), which indicates how close the traced contour is to the original contour by comparing center‐to‐point distances at points in every 5‐degree interval, shown in Figure 5. The CMI is derived by calculating the average of these distance measurements, providing a standardized metric to evaluate the precision of each contouring method. This approach allows for an objective comparison of the delineation accuracy across different techniques. To calculate the CMI, the following equation is used, where $\Delta r_{i}$ represents the deviation at each interval and *N* is the total number of intervals, with *N* equaling 360 when assessments are conducted at every 1 degree interval.

CMIΔ=1−1N∑iNΔriri

### 2.5 Dosimetric comparison

To further strengthen our dosimetric evaluation, three new abstract shapes have been added for additional testing. These shapes were used to calculate both the ARI and CMI, providing a more comprehensive assessment of O‐ACT's accuracy. We also performed dosimetric analysis for all three shapes. For each shape, we fabricated cutouts using three different methods: hand‐drawing, CT scanning, and O‐ACT. These cutouts were then used for dose calculations in the treatment planning system (TPS) (Eclipse Ver. 16.5.5., Varian, Palo Alto, CA). We calculated the percentage difference (% Diff.) between each method. From these three shapes, we selected one for dosimetric measurements. We fabricated cutouts for this selected shape using three different methods: hand‐drawing, CT scanning, and O‐ACT. Using these cutouts, we conducted actual measurements with a 9 MeV electron beam and 100 monitor unit (MU) irradiation. We measured MU and dose profiles (both in‐line and cross‐line) for each cutout, allowing for a direct comparison between the different methods in terms of dosimetric accuracy.

The CT scanning approach simulated an established clinical workflow for skin lesion treatment planning. Four shapes were defined on a phantom using scaled printouts. These skin facsimiles were delineated using radio‐opaque tape. Once scanned, the visualized target shapes were digitized in the TPS for electron block manufacturing. Converting the shapes into the printouts allowed both qualitative and quantitative assessment of their fidelity to the original outlines.

MU values, summarized in Table 1, were calculated using calculation TPS (Eclipse eMC 15.6). We also conducted a dosimetric measurement using a linear accelerator and the MapCheck 2 quality assurance (QA) device to compare dose profiles.

**TABLE 1:** Monitor units (MU) and the comparison with the percentage difference (%) for the abstract shapes.

	MU		
Shape	Hand‐drawing	CT scanning	O‐ACT
Abstract 1	100.73	100.60	101.03
Abstract 2	101.61	101.29	99.69
Abstract 3	100.88	100.90	100.46
% Diff. ± SD (O‐ACT vs. hand‐drawing)	−0.7% ± 0.9%		
% Diff. ± SD (O‐ACT vs. CT scanning)	−0.5% ± 0.8%		
% Diff. ± SD (hand‐drawing vs. CT scanning)	−0.1% ± 0.1%		

Abbreviations: CT, computed tomography; O‐ACT, Optical Automatic Contour Tracing; SD, standard deviation.

## 3 RESULTS

**FIGURE 6:**
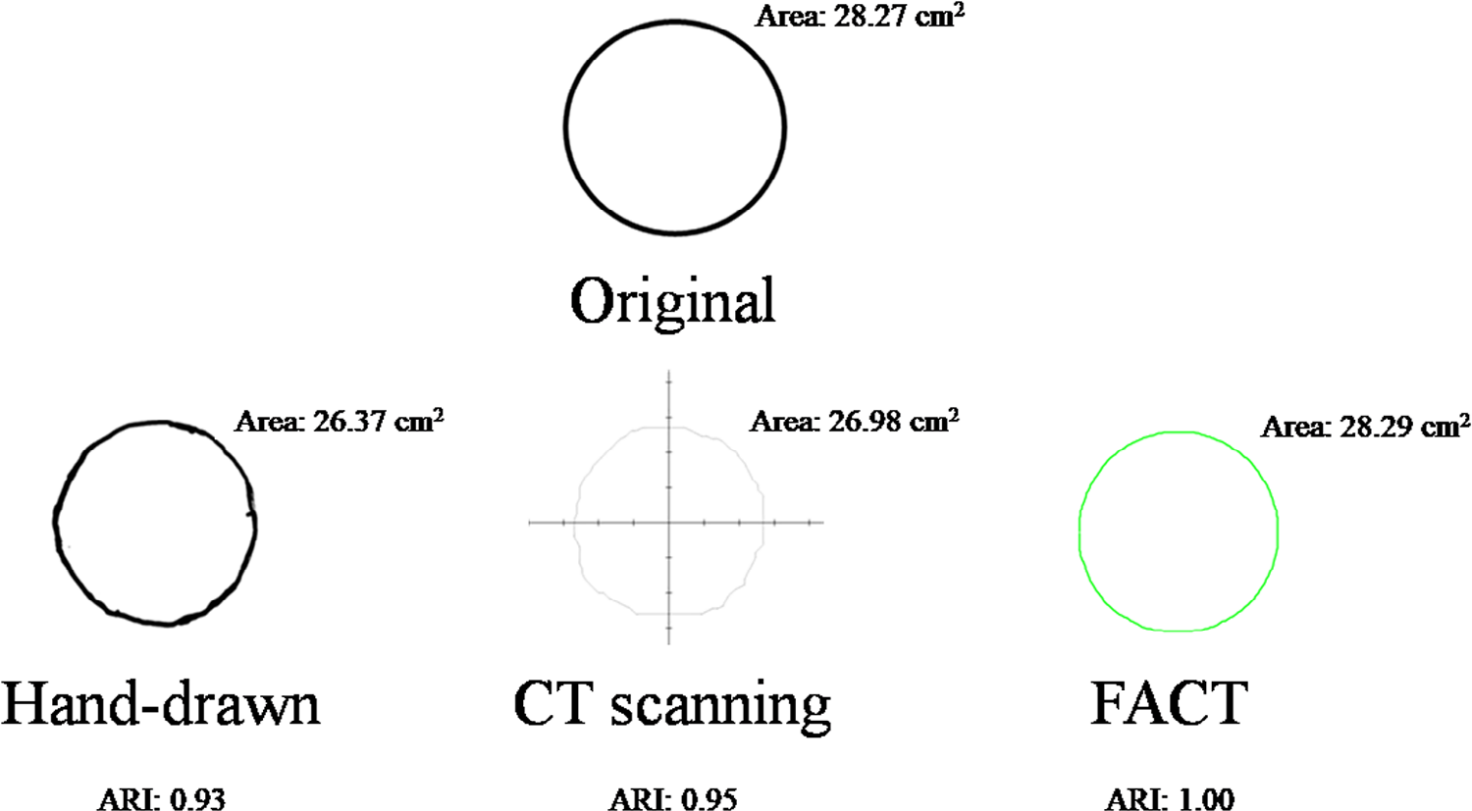
Comparative analysis of contour delineation methods versus the original contour. While the traditional hand‐drawing approach shows some deviations, the O‐ACT method, utilizing advanced image processing and magnification calculations, aligns closely with the original. The CT scanning, though sophisticated, presents slight variances when compared to the original contour. CT, computed tomography; O‐ACT, Optical Automatic Contour Tracing.

Figure 6 shows a representative result from various tests with different shapes. In this case, the original shape exhibited an area of 28.27 cm^2^. Using the traditional hand‐drawing method, the result was an area of 26.37 cm^2^ with an ARI of 0.93, suggesting noticeable deviations from the ideal. In contrast, the O‐ACT approach produced a contour area of 28.29 cm^2^ and an ARI of 1.00, indicating its closer alignment with the original. The CT scanning method registered an area of 26.98 cm^2^ and an ARI of 0.95. This evaluation highlights the accuracy and conformity of the O‐ACT method in comparison to both conventional techniques, as shown in Table 2. Overall, the O‐ACT showed the best performance, while the CT was the worst in terms of ARI.

**TABLE 2:** Comparison of the area (in cm^2^) and area ratio index (ARI) for various test shapes obtained from different methods: hand‐drawing, CT scanning, and O‐ACT.

	Original shape cm^2^	Hand‐drawing cm^2^ (ARI)	CT scanning cm^2^ (ARI)	O‐ACT cm^2^ (ARI)
Pear	19.80	22.94 (1.159)	19.60 (0.990)	19.98 (1.004)
Strawberry	24.10	23.24 (0.964)	22.63 (0.939)	24.02 (0.997)
Orange	28.27	26.37 (0.930)	26.98 (0.954)	28.29 (1.001)
Lemon	24.19	24.69 (1.023)	22.84 (0.944)	23.94 (0.990)
Abstract 1	17.23	15.58 (0.903)	17.95 (1.042)	17.35 (1.012)
Abstract 2	13.42	12.32 (0.924)	14.06 (1.052)	13.16 (0.984)
Abstract 3	16.97	17.87 (1.053)	16.13 (0.953)	16.58 (0.984)
ARI Mean ± SD		0.994 ± 0.084	0.982 ± 0.044	0.996 ± 0.010

*Note*: The mean and standard deviation (mean ± SD) for each method is presented at the bottom.

Abbreviations: ARI, area ratio index; CT, computed tomography; O‐ACT, Optical Automatic Contour Tracing; SD, standard deviation.

CMI values deviated from “1” more than ARIs did as expected. For example, the O‐ACT provided CMI values ranging from 0.93 to 1.00 (compared to 0.990 to 1.001 in ARI values). Table 3 summarizes CMI values obtained. Similar to the ARI results, the O‐ACT showed the best performance while the CT was the worst.

**TABLE 3:** Comparison of contour matching index (CMI) values for the test shapes using hand‐drawing, CT scanning, and O‐ACT methods, showcasing each technique's contour delineation precision.

Shape	Hand‐drawing	CT scanning	O‐ACT
Pear	0.916	0.859	0.956
Strawberry	0.908	0.909	0.931
Orange	0.969	0.937	0.996
Lemon	0.931	0.875	0.955
Abstract 1	0.941	0.956	0.974
Abstract 2	0.901	0.937	0.957
Abstract 3	0.924	0.950	0.977
Mean ± SD	0.927 ± 0.021	0.918 ± 0.035	0.964 ± 0.019

Abbreviations: CT, computed tomography; O‐ACT, Optical Automatic Contour Tracing; SD, standard deviation.

**FIGURE 7:**
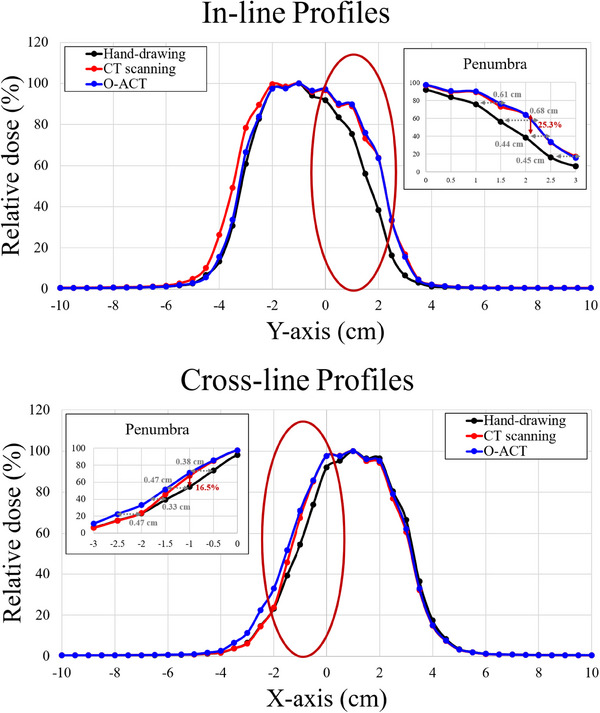
In‐line and cross‐line dose profiles for hand‐drawing, CT scanning, and O‐ACT methods. The insets highlight the penumbra regions, showing the distances between the two most divergent methods at specific relative dose percentages. These measurements quantify the maximum differences in penumbra width among the methods. CT, computed tomography; O‐ACT, Optical Automatic Contour Tracing.

Figure 7 illustrates the in‐line and cross‐line dose profiles for the selected abstract shape using the three methods.

In Figure 7, the insets highlight the penumbra regions (80% to 20%), showing the distances between the two most divergent methods at various dose percentages. Detailed analysis of the penumbra regions revealed significant differences among the methods. For the in‐line profile, the maximum distance difference in the penumbra region was observed at the 60% dose level, measuring 0.68 cm between the two most divergent methods. The largest dose difference was found at *y*‐axis position of 2 cm, where a 25.3% relative dose difference was observed. In the cross‐line profile, the penumbra region showed maximum distance differences of 0.47 cm at both the 20% and 60% dose levels. The most substantial dose difference was noted at *x*‐axis position of ‐1 cm, with a 16.5% relative dose difference between methods. These findings highlight the variability in dose distribution among the three methods, particularly in the critical penumbra regions. Table 1 presents the percentage differences in MUs for each method.

The differences in MU between the methods were minimal, with the largest difference of 0.68% observed between O‐ACT and hand‐drawing. CT scanning showed intermediate differences (0.54% vs. O‐ACT, 0.14% vs. hand‐drawing). These small differences suggest that all three methods provide consistent results, with O‐ACT showing a slight tendency to produce lower values. Combined with the dose profile analysis, these results indicate that O‐ACT offers comparable, if not slightly improved, dosimetric accuracy for the tested abstract shape, potentially enhancing precision in clinical dose delivery.

## 4 DISCUSSION

A new electron cutout generation method using optical imaging and its seamless workflow was introduced. It showed superior accuracy with straightforward data acquisition and processing than the current clinically accepted methods. CCD camera‐based optical imaging has many applications in radiation therapy. For example, Shandiz et al. applied the same distance calculation algorithm to the optical distance indicator (ODI) and gantry angle QA of the linear accelerator.^14^ And surface‐guided radiation therapy (SGRT) is also an advanced application of optical imaging to detect the deformation of the patient's surface.^15^

The O‐ACT method, while pioneering advancements in radiation therapy planning, faces unique challenges when pondering different approaches to image capture. One might suggest the intuitive solution of adjusting the height of the couch instead of altering the camera's position in the electron applicator. However, this seemingly straightforward alteration brings about several complications. First, even minor adjustments to the couch can induce unintended patient movements. These subtle shifts, particularly between two image captures, might introduce inaccuracies in the contour representation. Additionally, if the gantry is tilted at an angle other than 0 degrees, altering the couch position requires 2D displacements, making the process more complicated. Moving the couch also presents potential collision risks, especially in intricate configurations or in treatment rooms with spatial limitations. This adjustment might pose safety concerns and cause interruptions in the procedure. Given these considerations, the appeal of merely adjusting the couch is outweighed by the challenges, underscoring the significance and practicality of the O‐ACT method's approach of capturing images at different heights of the electron applicator.

A second concern is the universal applicability of the camera mounting mechanism. Given the variation in Linac designs among different manufacturers, such as Varian or Elekta, and differing electron applicator sizes, a custom holder design is necessary for each case. Any imperfections in the mount can result in minor angle changes during photo capture, possibly distorting the contour and compromising the treatment's accuracy. Despite these potential challenges, the O‐ACT method's benefits are clear. It removes a bulky camera setup to bring the focal spot to the source position and can simulate the patient at clinical gantry and couch positions using an electron cone compared to the previous study.^9^ It also provides significant convenience, especially in situations involving complex patient positioning with a gantry and a couch rotation, enabling more sustainable scheduling for oncologists. This improvement allows oncologists to see more patients without physical strain. Additionally, the final contour's PDF format facilitates easy adjustments. Oncologists can employ software such as Adobe Acrobat or Notability to swiftly correct minor inaccuracies and print the final design onto the cutout.

Another question that may arise is the rationale behind the shapes chosen for experimentation. We used both fruit shapes and abstract forms. These selections were made to encapsulate the seemingly random nature of tumor contours while still preserving an “organic” quality. Fruits were chosen for their naturally occurring irregular shapes, while abstract forms were used to represent the unpredictable patterns of tumor growth. This combination allows us to test our method on a range of shapes that mimic the diversity found in clinical scenarios.

In evaluating the methodology delineated in the M&M section, one of its notable attributes is its inherent adaptability. While this study employs 5‐degree intervals as a standard, it is highly adaptable to the intricacies of different shapes. For instance, a more complex contour might necessitate finer intervals (e.g., 1‐degree), whereas a simpler shape could be aptly captured with larger intervals. This flexibility not only speaks to the robustness of our approach but also ensures its applicability across a broad spectrum of scenarios.

An important consideration in our comparative analysis is the apparent inconsistency between the ARI/CMI data and the dosimetry results. Specifically, our method shows closer alignment with the hand‐drawing method in terms of ARI and CMI, while exhibiting greater similarity to CT scanning in dosimetry data. This discrepancy can be attributed to the inherent nature of these metrics. ARI and CMI are purely geometric measures, accounting for overall shape area without considering minor peripheral deviations. In contrast, dosimetry data and dose profiles are sensitive to these subtle edge variations, thus revealing differences not captured by ARI and CMI.

The clinical implications of the dosimetry comparisons are noteworthy. When evaluating output dose values in the TPS, we utilized contours generated by all three methods (hand‐drawing, CT scanning, and O‐ACT), as well as a reference contour representing the most accurate tumor delineation. Our findings indicate that the O‐ACT method produced results closest to the reference, while CT scanning and hand‐drawing methods showed greater deviations. To further validate these findings and demonstrate the efficacy of the O‐ACT method, we conducted a second dosimetry experiment generating dose‐profiles for comparative analysis. The O‐ACT method is designed to be effective across various lighting conditions and skin tones. Standard room lighting in the treatment area provides sufficient illumination for the camera to capture clear images, even on darker skin surfaces. Furthermore, our image processing algorithm, particularly the skeletonization step, enhances the contrast of the contour, ensuring clear delineation regardless of the patient's skin color. This approach allows for consistent performance across a diverse patient population.

Regarding the evaluation methods in this study, it is noted that CMI offers more accuracy than the ARI. The ARI, while useful, calculates average values and can potentially mask discrepancies. For instance, a significant underestimation at one point could be offset by an overestimation at another, leading to an averaged value that does not accurately reflect the precision of contouring. In contrast, the CMI, by evaluating discrepancies without a directional tradeoff, provides a more detailed and granular assessment of contouring accuracy, making it a more reliable metric for evaluating the precision of different contour delineation techniques.

Comparing our O‐ACT method with existing solutions like DotDecimal's cloud‐based service reveals several key advantages. While both aim to improve electron cutout creation, O‐ACT offers a direct beam's eye view, enhancing target visualization accuracy. It utilizes existing equipment in radiotherapy departments, minimizing additional costs. O‐ACT allows for immediate on‐site data interpretation and verification, enabling healthcare professionals to make quick decisions. Importantly, it can be implemented independently without relying on external services, making it particularly valuable for resource‐limited settings and developing countries. This independence, combined with its potential cost‐effectiveness in the long term, positions O‐ACT as a versatile solution that could democratize access to efficient electron therapy planning tools globally. As we continue to refine this method, we anticipate it playing a significant role in improving accessibility to accurate radiation therapy techniques across diverse healthcare settings.

In summing up our discussions, the O‐ACT method stands as an exemplar in radiation therapy planning. Its precision, combined with the rapidity of its process, minimizes the waiting time for patients and ensures accurate delineation. While the approach confronts specific challenges, the advantages it brings, such as adaptability and robustness, make it a prime choice in clinical settings. As we move forward in this field, the O‐ACT method's efficiency and versatility promise to enhance the workflow, ensuring both patients and practitioners benefit from its innovation. It is worthwhile to mention that it could be easily extended to the photon block fabrication for the patients or disease sites that are challenging with the CT simulation, like the total body irradiation (TBI).

## 5 CONCLUSION

It has been demonstrated the O‐ACT method could provide electron cutouts with better accuracy compared to two commonly used conventional techniques (the hand‐drawing and CT scanning based methods). It presents a transformative solution, leveraging optical capture and dedicated software to revolutionize custom block creation. This innovation ensures greater precision, streamlined processes, and improved patient care.

## CONFLICT OF INTEREST STATEMENT

The authors declare no conflicts of interest.

## Data Availability

The datasets used in this study are available from the corresponding author upon reasonable request.

## CALCULATION OF A CUTOUT COORDINATE FROM THE CAMERA PROJECTION

### 1

Place the camera at the lower and upper scrapers of the electron applicator using the mount. The distance between the camera's optical origin and the isocenter plane is already measured as *h*_1_ and *h*_2_ at each scraper. Take two pictures at each scraper: The upper image will be shown as smaller than the lower one. Contour shapes will be shown differently if they were drawn on a concave or convex surface.

From the symmetry in Figure 2a, the following relation is established between an arbitrary point *D(x*, *y*, *z)* on the patient's skin marking and a point *N_i_(x_i_*, *y_i_)* of the contour on the camera image at the *i‐th* image plane (e.g., *N*_1_ *(x*_1_ *, y*_1_) at the upper imaging plane and *N*_2_ *(x*_2_ *, y*_2_) at the lower imaging plane in the figure),

$$\;\frac{x\;\left(\mathrm{or}\;y\right)}{h_{1}+z}=\frac{x_{1}\left(\mathrm{or}\;y_{1}\right)}{d}\;\mathrm{and}\;\frac{x\;\left(\mathrm{or}\;y\right)}{h_{2}+z}=\frac{x_{2\;}\left(\mathrm{or}\;y_{2}\right)}{d}\;$$

where,

- *h*_1_, *h*_2_: Vertical distance between the optical origin and the isocenter plane
- *N_i_(x_i_*, *y_i_)*: An arbitrary point on the *i*‐th projection image obtained with the camera at *h_i_*
- *D(x*, *y*, *z)*: An arbitrary point on the skin marking
- *z*: Vertical distance between the point *D* on the skin marking and the isocenter plane
- *d*: Vertical distance between the optical origin and virtual imaging plane

By solving these regarding *z*, 3D coordinates (*x*, *y*, *z*) of the skin marking can be determined by

$$x_{1}\left(h_{1}+z\right)=x_{2}\left(h_{2}+z\right)$$

$$y_{1}\left(h_{1}+z\right)=y_{2}\left(h_{2}+z\right)$$

$$z=\frac{x_{1}h_{1}-x_{2}h_{2}}{x_{2}-x_{1}}=\frac{y_{1}h_{1}-y_{2}h_{2}}{y_{2}-y_{1}}$$

$$x=\frac{x_{1}x_{2}\left(h_{1}-h_{2}\right)}{d(x_{2}-x_{1})},\;y=\frac{y_{1}y_{2}\left(h_{1}-h_{2}\right)}{d(y_{2}-y_{1})}$$

Here, the distance *d* could be calculated independently by comparing the known dimension of an object, for example, a letter paper, placed on the couch to the captured object size in the camera at any scraper of the applicator:

$$d=h_{1}\;\frac{l_{1}}{L}=h_{2}\;\frac{l_{2}}{L}$$

where *L* is the known length of an object and *l*_1_, *l*_2_ are the captured sizes at the camera heights of *h*_1_, *h*_2_.

Now another symmetry is established between an arbitrary point *D(x, y, z)* on the skin marking and a point *B(x_B_, y_B_)* on the cutout about the radiation source as shown in Figure 2b:

$$\;\frac{x\;\left(\mathrm{or}\;y\right)}{SSD+z}=\frac{x_{B}\;\left(\mathrm{or}\;y_{B}\right)}{b}\;$$

where,

- *SSD*: Source‐to‐surface distance, that is, 100 cm
- *b*: Source to cutout tray distance, for example, 95 cm for a Varian linear accelerator
- *B(x_B_, y_B_)*: An arbitrary point on the electron cutout at the distance of *b*

So, the two‐dimensional (2D) coordinate of the cutout could be expressed as below using the 3D coordinate *(x, y, z)* of the skin marking determined in the previous step:

$$x_{B}\;\left(\mathrm{or}\;y_{B}\right)=\frac{x\;\left(\mathrm{or}\;y\right)\cdot b}{SSD+z}$$

$$\;x_{B}=\frac{x\cdot b}{SSD+z}\;\mathrm{and}\;y_{B}=\frac{y\cdot b}{SSD+z}\;$$

Therefore, the physical cutout coordinate could be calculated without knowing the actual depth information of the skin marking contour (i.e., *z*).
